# Effects of Glucocorticoids on Apoptosis and Clearance of Apoptotic Cells

**DOI:** 10.1100/tsw.2007.224

**Published:** 2007-08-17

**Authors:** Aisleen McColl, Sylwia Michlewska, Ian Dransfield, Adriano G. Rossi

**Affiliations:** MRC Centre for Inflammation Research, The Queen's Medical Research Institute, University of Edinburgh Medical School, 47 Little France Crescent, Edinburgh, EH16 4TJ, Scotland, UK

**Keywords:** glucocorticoids, apoptosis, inflammation, macrophage phagocytosis

## Abstract

The glucocorticoid (GC) drugs are one of the most commonly prescribed and effective anti-inflammatory agents used for the treatment of many inflammatory disorders through their ability to attenuate phlogistic responses. The glucocorticoid receptor (GCR) primarily mediates GC actions via activation or repression of gene expression. GCs directly induce the expression of proteins displaying anti-inflammatory activities. However, the likely predominant effect of GCs is the repression of multiple inflammatory genes that invariably are overexpressed during nonresolving chronic inflammation. Although most GC actions are mediated through regulation of transcription, rapid nongenomic actions have also been reported. In addition, GCs modulate inflammatory cell survival, inducing apoptosis in immature thymocytes and eosinophils, while delaying constitutive neutrophil apoptosis. Importantly, GCs promote noninflammatory phagocytosis of apoptotic cell targets, a process important for the successful resolution of inflammation. Here, the effects and mechanisms of action of GC on inflammatory cell apoptosis and phagocytosis will be discussed.

### INTRODUCTION

Inflammation is an important physiological host defence mechanism against infection and injury. Granulocytes, such as neutrophils and eosinophils, are crucial in innate immune defence against bacterial and parasitic infections, respectively[1]. However, the persistent recruitment and/or enhanced survival of granulocytes at inflamed sites may result from dysregulated expression of proinflammatory genes, such as cytokines (IL-1, TNFα, GM-CSF, etc.), chemokines (IL-8, IL-5, MIP-1α), and adhesion molecules (ICAM-1 and E-selectin)[2]. Uncontrolled leukocyte responsiveness will lead to release of inflammatory mediators, such as eicosanoids (prostaglandins, leukotrienes, and thromboxanes), cytokines (IL-8, etc.), reactive oxygen/nitrogen species ($O_2^-$, NO), and granular enzymes (e.g., elastase)[3], resulting in damage to the surrounding tissue and propagation of the inflammatory response. This likely contributes to persistent dysregulated inflammation, resulting in the pathogenesis of disorders such as chronic obstructive pulmonary disease (COPD) and bronchial asthma[4].

Apoptosis is a programmed form of cell death[5] that regulates the number and fate of leukocytes at inflamed sites and, in contrast to necrosis, is associated with maintenance of cell membrane integrity and damage limitation[6]. Granulocytes are terminally differentiated cells and undergo constitutive apoptosis during *in vitro* culture[6,7], which is amenable to lineage-specific modulation by intrinsic and extrinsic factors including cAMP[8], IL-5[7], GM-CSF[9], prostaglandins[10], and TNFα[11]. Synthetic glucocorticoids (GCs), such as dexamethasone, initially demonstrated to induce apoptosis of immature thymocytes[12], also accelerate apoptosis in eosinophils while, surprisingly, prolong neutrophil survival[13,14]. This observation has led to speculation that GCs may be exerting part of their beneficial effect in eosinophil-dominant diseases (e.g., asthma) by inducing apoptosis of lymphocytes and eosinophils while maintaining beneficial neutrophil-dependent antimicrobial responses. Consequently, it is possible that direct pharmacological triggering of cell-specific apoptosis may be a novel therapeutic strategy in the treatment of inflammatory disorders[15,16]. However, for this to be considered as a successful tactic, efficient removal of the apoptotic leukocytes is also required. Failure to do so may lead to apoptotic cells undergoing secondary necrosis with deleterious consequences in terms of tissue damage and the outcome of the inflammatory response. An additional anti-inflammatory effect of GCs is the profound potentiation of phagocytic clearance of intact apoptotic leukocytes described *in vitro*[17,18] and possibly *in vivo*[19]. These recently described effects of GCs, together with limiting inflammatory cell recruitment and activation, may prevent further injury through the release of noxious intracellular contents and may be important for promoting the resolution of inflammation. However, there are potential limitations to the application of GCs in disease, particularly as a consequence of the undesirable side effects associated with long-term treatment. Delineating the precise mechanisms of GC action would provide a significant insight into the anti-inflammatory role of GCs and allow the development of novel strategies that are more selective in their action.

### THE GLUCOCORTICOID RECEPTOR

GCs mediate most of their effects by binding to glucocorticoid receptors (GCRs). The GCR is a member of the nuclear receptor superfamily, which includes receptors for mineralocorticoids and sex hormones[20]. Alternative splicing of the *gcr* gene generates two or more GCR isoforms with distinct functions. Although GCRα is the predominant isoform and is responsible for GC binding, GCRβ is a c-terminally truncated variant that cannot bind GC or regulate transcription, but can form heterodimers with GCRα to modulate GCRα function[21,22]. Inactive GCR resides in the cytoplasm as part of a multiprotein complex, being bound to chaperone molecules including heat shock proteins (HSP90) and immunophilin[23]. HSP90 forms interactions with GCR in the c-terminal domain essential for maintaining the correct configuration of GCR and also masks a nuclear localisation signal to prevent translocation of the unoccupied GCR to the nucleus[24]. Upon binding of GC to GCR, these chaperone proteins dissociate from the GCR, unmasking the nuclear localisation signal required for the activated GC-GCR complex to translocate to the nucleus[24]. Here, GCR can directly or indirectly modulate the transcription of multiple target genes.

### MECHANISMS OF GC ANTI-INFLAMMATORY ACTION

#### Transactivation: Induction of Anti-Inflammatory Gene Expression

One mechanism whereby GCs can mediate their action is via direct binding of GCRs to DNA to increase the transcription of anti-inflammatory genes, a process known as transactivation (Table 1). Ligand-activated GCRs translocate to the nucleus and bind specifically to palindromic glucocorticoid-responsive elements (GREs) found in the promoter region of GC-responsive genes[25,26]. The central domain of the GCR contains two zinc fingers essential for GCR dimerisation and binding to GRE sequences[26,27], with a point mutation in the D-loop of the central domain abolishing transactivation[28]. Thus, transcription of GC-responsive genes requires GCRs to be in the homodimeric form. GCR "switching on" of gene transcription also requires the recruitment of specific transcriptional coactivator proteins, which are important for localised chromatin remodelling and stabilisation of the basal transcriptional machinery[29].

This mechanism directly modulates the expression of secretory leukocyte proteinase inhibitor (SLPI)[30], IL-1 receptor antagonist[29], and C1q [31]. Annexin I (or Lipocortin 1) is an additional GC-inducible protein thought to mediate many of the anti-inflammatory actions of GCs. This is confirmed by studies in deficient mice, which have altered expression of annexins, COX-2, and cPLA2; exaggerated responses to carrageenin- or zymosan-induced inflammation; and partial resistance to the anti-inflammatory effects of GCs[32]. Administration of exogenous annexin I confers anti-inflammatory activity in some models of inflammatory disease[33]. Conversely, infusion of annexin I antibodies neutralises the effect of annexin I and abrogates the anti-inflammatory activities of GCs[33]. It has been suggested that autoantibodies to annexin I may contribute to GC resistance and the pathogenesis found in inflammatory diseases where GCs may be used as a treatment, such as rheumatoid arthritis and systemic lupus erythematosus (SLE)[34]. Additionally, annexin I–derived peptides have been shown to mimic some of the anti-inflammatory effects of endogenous annexin I, including a role in phagocytic clearance of apoptotic cells[35]. GCs also enhance the expression of other anti-inflammatory proteins that may be important in switching off signalling pathways engaged during persistant inflammation. Mitogen-activated protein kinase (MAPK) phosphatase 1 (MKP-1) dephosphorylates and inactivates MAPKs, such as p38 MAPK and c-Jun terminal kinase (JNK), which contribute to enhanced expression of proinflammatory mediators[36]. The suppressive effect of GCs on MAPK signalling is impaired in MKP-1 knockout mice, which show enhanced expression of proinflammatory genes, including COX2, TNF$\alpha$ and IL-1[36]. Thus, the anti-inflammatory action of GCs via transactivation may be important in aiding the resolution of inflammation and in boosting innate immune defence through the induction of protective proteins. Nevertheless, it is doubtful that all the effects mediated by pleiotropic GCs could be explained by enhanced production of a small number of proteins with anti-inflammatory properties.

### Transrepression: Inhibition of Proinflammatory Gene Expression

Although a small number of genes can be regulated directly, many more genes are regulated indirectly by the GCR via suppression of gene expression, a process known as transrepression (Table 1). Originally, this repression was thought to result from homodimeric GCRs binding to putative negative GRE sites in the promoter regions of proinflammatory genes to switch off their transcription[37]. However, the majority of genes repressed by GCs do not contain negative GRE sequences; therefore, alternative mechanisms to DNA binding must be engaged.

Ligand-activated GCRs can "switch off" inflammatory gene expression by a DNA-independent mechanism through direct protein-protein interactions with activated NF$\kappa$B and AP-1 transcription factors[38,39]. Since NF$\kappa$B and AP-1 induce the transcription of multiple inflammatory and immune genes characteristic of various inflammatory disorders[40,41], this mechanism of GC action is extremely important and probably accounts for most of the inhibitory effects of GCs on inflammation. Although cytoplasmic interaction of ligand-activated GCR with transcriptional regulators has been reported, more recent data indicate that GCR may interfere with NF$\kappa$B and AP-1 at a later stage after they have bound to DNA to influence transcription[39,42]. Reichardt and colleagues introduced a point mutation A458T into the GCR gene by a gene-targeting technique involving Cre/loxP recombination[28,42,43]. Specifically, the mutation was "knocked in" to the D-loop of the central domain of the GCR, preventing DNA binding, GCR dimerisation (GCR$^{dim}$), and transactivation. However, transrepression of NF$\kappa$B- and AP-1–mediated gene expression remained intact, indicating that this process is mediated by monomeric GCR. Application of the GCR$^{dim}$ mouse model would allow investigation into the process of transrepression in the absence of transactivation and allow elucidation of GC mechanism of action in various processes during inflammation *in vivo*. Interestingly, higher concentrations of GCs are required for induction of anti-inflammatory gene expression, while gene repression by the GCR can occur at much lower, more clinically relevant concentrations of GC[24]. Indeed, it has been questioned whether DNA binding is required at all for physiological GCR function[44]. Thus, although GCs can induce anti-inflammatory gene expression, the repression of activated proinflammatory transcription factors appears to be the dominant mechanism of GC action. Moreover, these modes of GC effect may even occur concurrently, where the induction of IκBα and glucocorticoid-inducible leucine zipper (GILZ) may contribute to later onset inhibition of NFκB and AP-1, respectively[20].

**Table 1.**

**Mechanisms of Glucocorticoid Anti-Inflammatory Action**

| Mechanism of GC Action | Genes Affected |
|---|---|
| Transactivation: Induction of anti-inflammatory gene expression | SLPI, IL-1 receptor antagonist, C1q, annexin I, MKP-1, GILZ, IκBα |
| Transrepression: Suppression of proinflammatory gene expression | Cytokines (IL-1, TNFα, GM-CSF), chemokines (IL-8, MIP-1α), adhesion molecules (ICAM-1, E-selectin) |

### Chromatin Remodelling, HATs, and HDACs

It is now apparent that an additional DNA binding-independent mechanism of GC action may involve reversible alterations in chromatin structure and histone acetylation. Gene expression and repression are associated with alterations in chromatin structure through modification of core histones[45] (Fig. 1). In a resting cell, the DNA is tightly coiled around core histone proteins and is inaccessible to transcriptional regulators, a configuration referred to as “closed” and is associated with gene silencing[46]. Initiation of transcription by NFκB and AP-1 requires slackening of chromatin and unwinding of DNA for transcriptional cofactors and RNA polymerase II to gain access to genes. Upon binding to DNA, NFκB and AP-1 recruit and activate coactivator proteins, such as cAMP response element binding (CREB) binding protein (CBP) and p300/CBP-associated factor, which have intrinsic histone acetyltransferase (HAT) activity[46]. Acetylation of key lysine residues in histones by coactivator proteins initiates chromatin remodelling required for gene transcription[45]. Reversal of histone acetylation results in tightening of chromatin around DNA and hinders binding of NFκB and AP-1. Deacetylation is controlled by corepressor proteins, such as histone deacetylases (HDACs) and nuclear receptor corepressor (NcoR), and is associated with gene repression[29]. This process may be important for “switching off” genes once the stimulus is removed and inflammation is no longer required, and failure to do so could contribute to the development of persistent or chronic inflammation.

### GCR Interacts with HATs and HDACs to Regulate Their Function

GCR can directly interact with NFκB and AP-1 to suppress the expression of proinflammatory genes, a process that may involve reversal of histone acetylation[39] (Fig. 1). The exact mechanism involved here is uncertain, although a competitive role between GCR, NFκB, and AP-1 for binding to coactivators remains controversial[47]. GCRs may carry out their repressive function by directly suppressing the HAT

activity of coactivators[2]. Alternatively, activated GCRs may recruit corepressors, such as HDAC2, to the transcriptional complex to reverse histone acetylation[48].

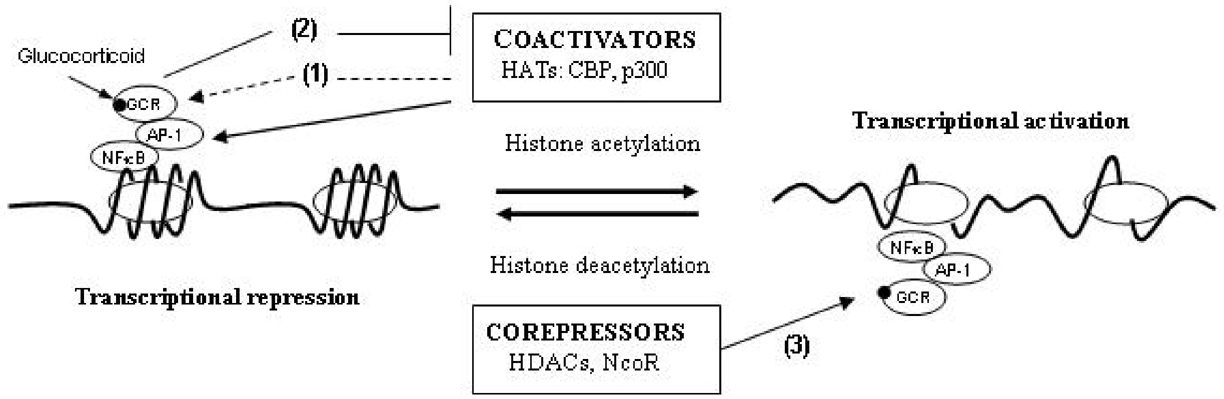

**FIGURE 1.** Effect of GCR on chromatin remodelling, HATs, and HDACs. Gene expression and repression are regulated by modification of core histones. In the resting state, DNA is tightly coiled around histones and is inaccessible to transcriptional cofactors and RNA polymerase II. Upon binding to DNA, NFκB and AP-1 recruit and activate coactivator proteins, such as CBP and p300/CBP-associated factor, which have intrinsic HAT activity. Histone acetylation results in slackening of chromatin and unwinding of DNA, allowing the transcriptional machinery to gain access to genes and initiate transcription. Deacetylation is controlled by corepressor proteins, such as HDACs and NcoR, and is associated with gene repression. Activated GCRs may interact with HATs and HDACs to regulate their function. Suggested mechanisms of GR transrepression include (1) GCR competes with NFκB and AP-1 for binding to coactivators, (2) direct suppression of HAT activity of coactivators by GCR, (3) recruitment of corepressors by GCR to reverse histone acetylation.

### Nongenomic Effects

The genomic effects of GCs have a lag period of around 30 min to a few hours/days[20], result from GC binding to cytoplasmic GCR, and are blocked by inhibitors of transcription and translation, such as cycloheximide[49]. Some data indicate that GCs may exert more rapid actions (seconds to minutes) that are most likely transcriptional independent[50]. Furthermore, it has been suggested that some immediate GC effects may be GCR independent, for instance, inhibition of superoxide production by macrophages[51] or alteration of plasma membrane properties[50]. Other investigations have implicated GCR-dependent nongenomic effects of GCs via post-transcriptional regulation, both at the level of mRNA and protein[52]. This may provide an immediate inhibitory effect of GCs on the expression, synthesis, and release of proinflammatory mediators. For this, a putative membrane-associated form of the GCR (mGCR) may bind GC and transduce signals via MAPKs, PI-3K, and PKC, which mediate the rapid effects[49,53]. Investigations by Croxtall and colleagues have implicated GCR-dependent effects of dexamethasone via altered phosphorylation status of annexin I, leading to inhibition of cPLA2 activation and arachidonic acid release[54]. Whether any of these nongenomic effects described occur at physiological concentrations of GC and have a contributory role to the anti-inflammatory effect of these steroids remains controversial.

### GC RESISTANCE

GCs are pleiotropic steroids with the ability to attenuate many phlogistic responses, making them the most effective clinical treatment for various inflammatory disorders, e.g., asthma. However, a small proportion of asthmatic patients do not respond well to GC treatment and may require high doses of GC[55]. Several mechanisms have been postulated to contribute to GC resistance. Increased expression of GCRβ has been suggested to interfere with GCRα function[21,22]. Interestingly, the level of GCRβ is increased by proinflammatory cytokines[56] and in peripheral blood mononuclear cells from patients with GC-resistant rheumatoid arthritis[57]. The level of intracellular GC is much lower than the extracellular concentration due to expulsion of GC from the cell by members of the ABC family of transporter molecules[52]. Overexpression of these proteins may contribute to the development of GC resistance. COPD is an inflammatory disorder where GCs are not usually a beneficial treatment and patients are relatively resistant to these steroids even when given high doses[24]. This ineffectiveness has been linked to factors such as smoking and oxidative stress[58].

### GC SIDE EFFECTS

Despite being the mainstay therapy for treatment of inflammatory disease, long-term GC use is associated with many undesirable side effects, the molecular mechanisms of which are extremely complex and are not entirely understood. Endocrine and metabolic side effects leading to diabetes and osteoporosis have been linked to transactivation of genes by the ligand-activated GCR[59]. For example, GCs may increase glucose synthesis through transactivation of enzymes involved in gluconeogenesis[59]. $GCR^{dim}$ mutants, which are transactivation deficient, retain many of their beneficial anti-inflammatory effects through transrepression of NFκB- and AP-1–mediated gene expression[28,42,43]. Hence, identification of "dissociated steroids" that can mediate transrepression of proinflammatory genes without induction of transactivation would represent an attractive therapeutic strategy. Such compounds may provide a safer approach to treatment of inflammatory diseases through this ability to convey the beneficial actions of GCs with reduced adverse effects[60]. However, some side effects, such as skin atrophy and suppression of the hypothalamic-pituitary-adrenal axis, may be mediated by the transrepressive function of the GCR[59] or may even involve a combination of both GCR mechanisms that would complicate the use of dissociated steroids in therapy. It is imperative, therefore, that the specific molecular mechanisms involved in GC-induced side effects are delineated to allow the development of more selective therapies.

### GCS AS A THERAPY FOR INFLAMMATORY DISEASE

#### Inflammation is Normally Self-Resolving

Under normal circumstances, inflammation is beneficial to the host and is self-resolving, requiring controlled clearance of granulocytes from inflamed sites[6]. Apoptosis of granulocytes, in contrast to necrosis, is associated with maintenance of membrane integrity[6] and down-regulation of potentially injurious secretory responses[61]. For granulocytes, functional attenuation is also achieved by surface changes during the apoptotic process, including down-regulation of FcγRIII (CD16)[62] and L-selectin expression[63], and uncoupling β2 integrins[63]. Cells undergoing apoptosis display specific surface alterations important for signalling rapid recognition and internalisation by phagocytes[64,65,66]. Many receptor mechanisms have been acknowledged in the direct recognition and engulfment of apoptotic cells by phagocytes[67,68,69], including the vitronectin receptor[70], scavenger receptors[71], CD31[72], putative phosphatidylserine receptor (PSR)[73], C1q receptor calreticulin[74], and the tyrosine kinase receptor, MER[75]. Blocking individual receptors on phagocytes only partially inhibits phagocytosis of apoptotic cells, suggesting redundancy in the engulfment pathways and underlining the importance of phagocytic clearance of apoptotic cells. During resolving inflammation, macrophages (or other cells with phagocytic capacity) are required to remove the potentially histotoxic apoptotic cells rapidly and efficiently[76]. Phagocytic removal of apoptotic cells normally occurs early on in the death process when the dying cell is still intact[6] and, in contrast to phagocytosis of necrotic cells, fails to induce the release of proinflammatory mediators, such as eicosanoids, GM-CSF, or IL-8, and MCP-1 chemoattractants from the phagocytic cell[77,78,79]. Engulfment of apoptotic cells also actively induces anti-inflammatory and immunosuppressive effects in the phagocyte by, for example, inducing the production of IL-10, TGFβ, PGE2, and platelet-activating factor[80,81]. Thus, rapid recognition, ingestion, and degradation of dying cells by phagocytes are nonphlogistic and equally as vital as the process of apoptosis itself. Both apoptosis and subsequent phagocytic clearance are prerequisites for efficient clearance of granulocytes and other inflammatory cells from tissues and, ultimately, for the resolution of inflammation.

#### Disease: Failure of Natural Resolution Process?

Neutrophils and eosinophils have been implicated in the pathogenesis of many inflammatory and allergic disorders, such as COPD and bronchial asthma. Accumulation of leukocytes reflects a mismatch between their infiltration into the inflamed site in response to cytokines, chemokines, and adhesion molecules, which are overexpressed during chronic inflammation and the mechanisms required for their clearance. Defective apoptosis or failure to remove intact apoptotic cells efficiently before lysis may play a contributory role in the disease pathogenesis through release of histotoxic granulocyte contents that have the capacity to damage the surrounding tissue and by stimulating proinflammatory macrophage secretory responses. Indeed, impaired phagocytosis of apoptotic cell material may contribute to the severity of disease in human SLE[82,83]. Mouse models of C1q deficiency develop spontaneous/persistant inflammation that resembles an SLE-like disease and may be due to defective clearance of apoptotic cells[84,85]. In both cases, a systemic autoimmunity is developed against autoantigens that may be displayed on or released by the noningested apoptotic cells. This underlines the importance for “safe” and efficient removal of these potentially harmful cells and the therapeutic value of agents that promote this. Our laboratory and others have identified GCs as important regulators of both apoptosis and phagocytic removal of apoptotic cells in diseases where these processes may be inefficient or dysregulated. Indeed, it has recently been demonstrated that manipulation of apoptosis *in vivo* may result in enhanced resolution of inflammation[86,87], thereby highlighting the potential to manipulate processes involved in the resolution of inflammation for therapeutic gain[15,16]. Consequently, the effects of glucorticoids on the resolution process, in our opinion, are highly clinically relevant.

### GLUCOCORTICOID REGULATION OF APOPTOSIS IN INFLAMMATORY CELLS

#### Internal Controls of Apoptosis

Apoptosis plays a crucial role in regulation of the inflammatory response. Neutrophils have a short life span with a circulating half-life of 6–10 h, which is delayed by redundant mediators present at inflamed sites (24–48 h)[88]. Eosinophil apoptosis occurs at a much slower rate than in neutrophils[7]. Despite their derivation from a common precursor cell, apoptosis in these granulocytes is controlled quite differently. Many of the inflammatory mediators present at an inflamed site can exert either anti- or proapoptotic influences. For example, G-CSF, GM-CSF, C5a, and LPS profoundly inhibit neutrophil apoptosis[14,89,90], as does increased cAMP[8] or a hypoxic environment[91]. Eosinophil survival is promoted in response to GM-CSF, IL-3, and IL-5[92], and $PGD_2$[10].

T cells are an essential component of the adaptive immune system, and apoptosis is important for their development and maintenance. Apoptosis is fundamental in regulating T-cell development in the thymus during selection of developing thymocytes bearing a functional TCR[93]. Regulation of apoptosis in T cells differs from that in granulocytes. Thymocytes undergo apoptosis upon elevation of intracellular calcium concentrations in response to calcium ionophores[94], whereas increased levels of intracellular calcium delays eosinophil apoptosis[10]. Additionally, cycloheximide accelerates the constitutive rate of apoptosis in both neutrophils[95] and eosinophils[13], but not T cells[4]. Apoptosis is also essential for maintenance of mature T cells in the periphery. Upon encounter with cognate antigen, mature T cells undergo significant clonal expansion and removal of these expanded cells once the stimulus has been cleared is by activation-induced cell death[93], which involves activation of the extrinsic pathway of apoptosis by ligation of death receptors such as FasL[96]. Hence, apoptosis is an indispensable process to avoid autoimmunity.

From the knowledge gained on the internal controls of apoptosis, it may be possible to induce apoptotic death of specific inflammatory cells involved in the pathogenesis of diverse inflammatory diseases. In support of this suggestion, *in vitro* studies suggest that apoptosis in the closely related neutrophil and eosinophil granulocytes appears to be controlled by different mechanisms.

### GC Modulation of Apoptosis

Physiological concentrations of GCs can regulate the intrinsic rate of apoptosis in many leukocytes and their efficacy depends upon a number of modulatory factors, including the GC type and concentration. GC regulation of granulocyte survival is dose dependent and occurs through the GCR (blocked by mifepristone)[13,14]. The GCs dexamethasone, methylprednisolone, and hydrocortisone all prolonged neutrophil viability from 12 to 48 h, however non-GC progesterone failed to inhibit development of apoptosis[14,97], indicating this is not due to nonspecific effects of high dose GCs. Endogenous cortisone also mediates these effects on granulocyte survival, albeit to a lesser extent[13]. Differential effects of GCs on granulocyte apoptosis have also been shown in a rat peritoneal model[98], where various GCs induce eosinophil apoptosis, but delay neutrophil apoptosis with most significant effects observed at GC concentrations of $10^{-6}$ and $10^{-8}$ *M*, respectively. Thus, GCs used at optimal concentrations for eosinophil apoptosis prolong neutrophil survival, indicating that the beneficial effects of GC treatment for asthma may involve suppressing eosinophilic airway inflammation while maintaining antimicrobial responses. This is a more specific therapeutic approach to treatment of inflammatory diseases compared to general nonspecific immunosuppressive agents, such as cyclosporine. However, the anti-inflammatory action of GCs is not reproducible for COPD, a neutrophil-dependent disease. Here, GC-mediated neutrophil survival may contribute to tissue damage with increased potential for release of intracellular contents. NFκB plays a vital role in the regulation of granulocyte apoptosis by inducing the expression of prosurvival genes[99] and targeted inhibition may be an approach to induce neutrophil apoptosis in COPD. Indeed, the NFκB inhibitor gliotoxin induces apoptosis in granulocytes alone or synergistically with TNFα[99].

GCs can also induce apoptosis in thymocytes[12] and, hence, are used in the treatment of lymphoid diseases. Interestingly, thymocyte sensitivity to GCs seems to be dependent upon their developmental stage where GCs induce apoptosis in $CD4^+CD8^+$ double positive thymocytes, but not upon maturation to single positive thymocytes[93]. In contrast, mature T cells are relatively resistant to GC-induced apoptosis, and some investigations have even reported GCs to prevent activation-induced cell death in these cells[100]. The effect of GCs on monocyte viability is more controversial. Although GCs have been suggested to induce apoptosis in monocytes[101], a recent study by Ehrchen et al. showed that monocytes were protected from staurosporine-induced apoptosis, mediated by reactive oxygen species (ROS), by increasing the expression of antioxidant molecules upon GC treatment[102].

GC influence on cell survival is initiated by GC binding to the cytoplasmic GCR and involves alterations in gene expression. However, there has been much controversy over the mechanism used by the GCR, whether by transactivation of death genes, transrepression of survival genes or both.

## MECHANISMS OF GC EFFECTS ON APOPTOSIS

### Evidence for Transrepression

Helmberg and colleagues[103] have suggested that interference with proinflammatory signalling through the transrepressional activity of GCR is an important mechanism of GC-induced apoptosis. This was derived from studies using T-cell leukaemia cell lines expressing mutant GCRs that were DNA-binding and transactivation defective, but retained the ability to repress NFκB and AP-1 transcriptional capability. These mutants were sufficient to induce apoptosis in these cells in response to GCs, implicating a role for GCs in apoptosis via the transrepression of prosurvival genes. Transrepression by GCR is suggested to be the main mechanism of suppressing the expression of cytokines and other inflammatory mediators that modulate granulocyte survival, and this may contribute to GC-induced apoptosis. In a recent study, Novac and colleagues provide data that support the role of GCR repression of gene expression in GC-induced apoptosis[104]. Here, GCs transiently suppress the level of the death receptor FasL and subsequently inhibit activation-induced T-cell apoptosis. Regulation of FasL expression was suggested to be a result of GCR binding directly to DNA, specifically to negative GREs that overlap an NFκB site. Hence, repression of FasL by GCR binding *in cis* may be due to competition for a common binding site and their rationalisation of this mechanism is that it would slow down T-cell apoptosis so that macrophages are not overwhelmed with a massive apoptotic cell load.

### Evidence for Transactivation

Ramdas and Harmon[105] used a transactivation-defective mutant GCR that retained transrepressional activity, but did not mediate GC-induced apoptosis in human leukaemic T cells, suggesting that DNA binding by the GCR is important. Similarly, Reichardt and coworkers used a $GCR^{dim}$ mutant to show the requirement for transactivation in GC-induced thymocyte apoptosis[28]. New protein synthesis has been implicated in the regulation of apoptosis and, indeed, GC-induced thymocyte apoptosis and neutrophil survival can be abolished with cycloheximide[106]. This indicates that neutrophil survival requires ongoing gene expression and continuous synthesis of a prosurvival factor(s), a process that requires continued presence of GC[106], whereas GC-induced thymocyte apoptosis may be due to transactivation of death genes. Alternatively, GC induction of IκB expression may block proinflammatory signalling and contribute to induction of apoptosis in responsive cells[105].

### Effects of GCs on the Mitochondrial Pathway–Regulated Apoptosis

Regulation of apoptosis may occur at the level of the mitochondria, an intracellular organelle with a central role in the apoptotic process. During the early stages of apoptosis, loss of mitochondrial membrane potential and increase in outer mitochondrial membrane permeability allow the release of factors such as Cytochrome C[8], which initiates downstream effects culminating in caspase activation and ultimately apoptosis[107]. Human eosinophils stimulated with dexamethasone show morphological changes characteristic of apoptosis, including DNA fragmentation, caspase-3 activation, and loss of mitochondrial membrane permeability[107]. Inhibition of mitochondrial permeabilisation, but not caspase-3 activity, prevented both mitochondrial disruption and apoptosis in response to dexamethasone. This suggests that GC-induced eosinophil apoptosis occurs through activation of the mitochondrial pathway, possibly resulting from the release of caspase-independent apoptosis-inducing factor (AIF)[107]. Loss of mitochondrial membrane potential and induction of eosinophil apoptosis may result from oxidant-induced mitochondrial injury, a process enhanced by GCs through production of ROS and sustained activation of proapoptotic JNK[3]. Ruiz and coworkers used the glucose-glucose oxidase system to achieve a constant production of hydrogen peroxide as a source of ROS to stimulate oxidative stress in neutrophils and, hence, apoptosis[108]. Dexamethasone delayed ROS-induced apoptosis in a concentration-dependent manner, with most significant inhibition at $10^{-6}$ *M* dexamethasone at 12 and 24 h time points[108].

Although some studies described above do not implicate a role for caspases, GC-induced apoptosis in thymocytes and lymphoma cells can be blocked by pharmacological inhibition of caspases (z-VAD-fmk)[109], showing an additional level of apoptosis regulation, at least in these cell types, by GCs.

#### Involvement of Bcl-2 Family Members in GC-Induced Apoptosis

Bcl-2 proteins play a crucial role in apoptosis by regulating mitochondrial membrane stability[88]. Neutrophils express proapoptotic (Bak and Bax) and antiapoptotic (A1 and Mcl-1) members of the bcl-2 family[88]. GCs may delay neutrophil apoptosis resulting from mitochondrial dysfunction by increasing the abundance of antiapoptotic A1 mRNA, while decreasing the level of proapoptotic Bak mRNA in a GCR-dependent manner[88]. GC-induced thymocyte apoptosis may also result from mitochondrial dysfunction[110] as a consequence of alterations in the levels of pro- and antiapoptotic bcl-2 factors. Indeed, thymocyte apoptosis in response to dexamethasone is accelerated by bcl-2 deficiency[111], whereas bcl-2 overexpression inhibits GC-induced lymphoma cell apoptosis[112]. Furthermore, alterations in the phosphorylation status of bcl-2 family members in response to GCs may lead to mitochondrial injury and subsequent eosinophil apoptosis[3]. Thus, the intrinsic mitochondrial pathway may contribute significantly to determining the fate of cells by integrating signals from pro- and antiapoptotic members of the bcl-2 family of proteins and this may be regulated by GCs. This apoptotic pathway is also inhibited in human neutrophils by a cell permeable analogue of cAMP, dibutyryl cAMP (db-cAMP), resulting in neutrophil survival[8], whereas proinflammatory mediators G-CSF and GM-CSF increase the expression of proapoptotic bcl-2 proteins and, hence, may promote apoptosis[88].

### MODULATION OF PHAGOCYTIC REMOVAL OF APOPTOTIC CELLS BY MACROPHAGES

In situations where cells are stimulated to undergo apoptosis at a high rate (for example, when there are overwhelming proapoptotic signals), the tissue load of apoptotic cells may be in danger of exceeding the removal capacity by phagocytes and noningested apoptotic cells may then undergo secondary necrosis with detrimental consequences[113]. Thus, the phagocytic clearance of cells dying by apoptosis plays a pivotal role in determining the inflammatory outcome and may be a prerequisite for effective resolution of inflammation. Hence, in order for deliberate induction of apoptotic cell death to be considered as a therapeutic strategy, parallel strategies for their efficient removal will also be required.

#### Pharmacological Modulation

Our laboratory is interested in the way inflammation resolves and we have made attempts to modulate macrophage phagocytic potential via pharmacological means. Increased levels of cAMP in human monocyte-derived macrophages (MDMϕ), using db-cAMP, specifically inhibits phagocytosis of apoptotic neutrophils[114], whereas ligation of macrophage surface CD44 rapidly and specifically augments phagocytic uptake of apoptotic neutrophils, but not apoptotic lymphocytes[115]. Adhesion to the matrix protein fibronectin also rapidly increases macrophage capacity for internalisation of apoptotic neutrophils[116]. Particular focus is being made now on interpreting the influence of GCs on the mechanisms by which acute inflammation normally resolves. We have demonstrated that GCs augment phagocytic potential for nonphlogistic clearance of apoptotic leukocytes, a process that is essential for the resolution of inflammation. Pretreatment of 5-day human MDMϕ for the first 24 h of culture with GCs methylprednisolone, dexamethasone, and hydrocortisone induced a prophagocytic phenotype displaying augmented phagocytosis of neutrophils undergoing apoptosis[17]. GCs also enhance macrophage capacity for uptake of other apoptotic targets, including Jurkat T cells and eosinophils, and also promote uptake of apoptotic neutrophils by alternative phagocytes, including human glomerular mesangial cells. GC action on phagocytosis is specific, as non-GCs aldosterone, estradiol, and progesterone did not exert this effect and GCs did not promote ingestion of Ig-opsonised erythrocytes. These observed effects are GCR dependent, being inhibited by the GCR antagonist mifepristone, and are reversed by cycloheximide, indicating the requirement for new protein synthesis.

### Reprogramming of Monocyte Differentiation by GCs

Freshly isolated monocytes lack the capacity to ingest apoptotic targets, an ability that is gradually acquired during *in vitro* culture as adherent monocytes differentiate into macrophages[117]. GCs potentiate the phagocytic capacity of MDMϕ cultured for 5 days *in vitro*, with a greater potentiation observed the earlier monocytes were exposed to GCs during maturation[17]. Furthermore, we have demonstrated that long-term exposure (5 days) of monocytes to GCs induced differentiation of monocytes into MDMϕ displaying enhanced phagocytosis of apoptotic cells (up to threefold) and, hence, represents a proresolution phenotype[18]. Macrophages that had been treated with GC for this period were a more homogenous population of smaller, more rounded, and less well-spread cells with a phenotype characterised by reduced phosphorylation and, hence, recruitment of paxillin and pyk2 to sites of adhesion, with consequential loss of actin- and paxillin-containing podosomes, and loss of p130cas expression, an important adaptor molecule in integrin adhesion signalling through the DOCK180/Crk/p130cas pathway. Despite their altered adhesion, time-lapse video microscopy revealed that GC-treated MDMϕ remained membrane active with extension and retraction of cellular process, probably as a result of increased levels of active Rac. GC-treated MDMϕ also displayed homogeneity in surface receptor expression, as shown by their laser scatter properties from flow cytometry, which identified a more uniform expression of HLA-DR, CD14, and CD44, consistent with reprogramming during monocyte differentiation. Although GC-treated MDMϕ exhibit elevated surface expression of the haemoglobin scavenger receptor, CD163, no single surface receptor was identified that would define a prophagocytic macrophage phenotype. Combinatorial treatment with GC and the “classical activator” IFNγ abolished GC potentiation of macrophage phagocytic capacity for apoptotic cells[118]. The first 24 h of culture with GC was critical for acquisition of a prophagocytic phenotype as this was overridden by subsequent treatment with IFNγ, with less pronounced inhibition the later IFNγ was added. Interestingly, IFNγ did not have an observed effect on GC-mediated morphology or surface receptor expression indicating that GC-treated MDMϕ adhesion status can be dissociated from phagocytic capacity. This has implications for the application of GC therapy in Th1-mediated diseases characterised by high levels of IFNγ.

### Induction of an Anti-Inflammatory Macrophage Phenotype by GCs

GCs are associated with induction of an anti-inflammatory macrophage phenotype. Importantly, GC potentiated phagocytosis of apoptotic cells did not stimulate the release of proinflammatory mediators, including MCP-1 and IL-8 chemokines[17]. Therefore, GCs promote “safe” clearance of cells dying by apoptosis and may directly contribute to the resolution of inflammation. The GC-inducible protein annexin I undergoes caspase-dependent recruitment from the cytosol to the outer plasma membrane during apoptosis to colocalise with phosphatidylserine and may be required for efficient clearance of dying cells[33]. Alternatively, GC-induced expression of annexin I in macrophages and subsequent release may enhance apoptotic cell uptake[35]. Indeed, macrophages from annexin I knockout mice show defective phagocytosis of apoptotic cells acting through the FPRL1[35]. This has been furthered by a recent study by Scannell et al., who report that annexin I is a prophagocytic factor released by apoptotic cells and actively promotes the FPRL1-dependent clearance of apoptotic cells by macrophages[119]. Thus, transactivation of annexin I expression by the GCR may contribute to enhanced recognition and internalisation of apoptotic cells in response to GC treatment.

C1q is an important subcomponent of complement C1, which activates the classical pathway upon binding to immune complexes or CRP and its expression in monocytes/macrophages can be induced by GCs or inhibited by IFNγ and LPS[31,120]. C1q binds to apoptotic cells[121] and may aid in their removal by phagocytes. Interestingly, Botto and colleagues demonstrated C1q deficiency in mice to cause an SLE-like disease with high titres of autoantibodies and accumulation of apoptotic cells in glomeruli[85] and defective clearance of apoptotic cells is also observed in human SLE patients[82,83]. Thus, C1q may protect against development of SLE by targeting apoptotic cells for clearance. This has been supported by *in vitro* studies where C1q-deficient human macrophages show impaired phagocytosis of apoptotic cells, and this is restored with purified human C1q[84]. Hence, induction of innate proteins by GCs may contribute to their anti-inflammatory effect by enhancing removal of dying cells.

A recent study by Ehrchen and coworkers used a microarray system to analyse the expression profile in human monocytes treated with GCs[102]. Their data signify the multitude of GC effects, where the expression of over 100 genes were GC regulated with a more pronounced induction of genes that are important in monocyte/macrophage functions such as phagocytosis, apoptosis, and adhesion. Important to the process of phagocytosis, there was induced expression of CD163, FPR1, and MER tyrosine kinase receptors, and MFG-E8 and C1q serum proteins, however, the relevance of this in GC-potentiated phagocytic capacity is unclear. These results indicate that GC effects on monocytes are not simply immunosuppressive, but also immunomodulatory. GCs promote induction of an anti-inflammatory phenotype important for the resolution of inflammation, challenging the concept that transrepression is the dominant mechanism of GC action on monocytes.

### CONCLUSION

Apoptosis is a fundamental process in cell and tissue homeostasis that, in contrast to necrosis, is associated with maintenance of cell membrane integrity and noninflammatory clearance by phagocytes. Failure or inefficient apoptosis and/or phagocytosis may result in necrosis with detrimental proinflammatory consequences. GCs modulate inflammatory cell survival and promote nonphlogistic phagocytosis of apoptotic cell targets *in vitro*, a situation that could be deemed proresolution with consequent implications in the clinical setting where unresolving chronic inflammatory diseases cause considerable morbidity and untimely death. Thus, considerable effort to elucidate the precise molecular and cellular mechanisms of action of GCs, as well as improve existing GCs, is being made.

### ACKNOWLEDGEMENTS

We thank the Medical Research Council UK (MRC), The Wellcome Trust, and the Arthritis Research Campaign (16140) for financial support throughout recent years. AM is an MRC-funded PhD student and SM is a Wellcome Trust–funded PhD student.
